# No evidence for a signal in mammalian basal metabolic rate associated with a fossorial lifestyle

**DOI:** 10.1038/s41598-024-61595-1

**Published:** 2024-05-17

**Authors:** Hana N. Merchant, Jack E. Thirkell, Steven J. Portugal

**Affiliations:** 1 Department of Biological Sciences, School of Life and Environmental Sciences, Royal Holloway University of London, Egham, Surrey TW20 0EX UK; 2 Department of Biology, University of Oxford, OX1 3SZ Oxford, United Kingdom

**Keywords:** Ecophysiology, Evolutionary ecology

## Abstract

A vast array of challenging environments are inhabited by mammals, such as living in confined spaces where oxygen levels are likely to be low. Species can exhibit adaptations in basal metabolic rate (BMR) to exploit such unique niches. In this study we use 801 species to determine the relationship between BMR and burrow use in mammals. We included pre-existing data for mammalian BMR and 16 life history traits. Overall, mammalian BMR is dictated primarily by environmental ambient temperature. There were no significant differences in BMR of terrestrial, semi-fossorial and fossorial mammals, suggesting that species occupying a subterranean niche do not exhibit baseline metabolic costs on account of their burrowing lifestyle. Fossorial mammals likely show instantaneous metabolic responses to low oxygen in tunnels, rather than exhibit adaptive long-term responses in their BMR.

## Introduction

Metabolic rate—the amount of energy expended by an animal over a specific period of time—is often referred to as the currency of life^1,2^. Understanding energy expenditure and energetic budgets is an important component, therefore, in explaining how species are adapted to their environment and any associated niche partitioning^3^. Moreover, metabolic rate can be used to predict the environmental conditions, such as temperature and elevation, that a species can tolerate^4,5^. Metabolic rate can also be used to determine aspects of an organism’s thermal neutral zone (TNZ), which is the range of temperatures at which the metabolic rate of an organism is at its minimum in response to ambient temperature^6^.

Basal metabolic rate (BMR) is a standardised measure, whereby metabolic activity is measured in non-lactating, non-reproductive, post-absorptive adults, whilst individuals are at rest during their natural rest phase of a 24-h cycle and within their thermoneutral zone^7^. BMR can, however, change throughout the annual cycle, exhibiting a degree of plasticity. Such plasticity enables organisms to alter their BMR in response to certain life-history events^8–10^. Natural environmental changes, such as seasonal variability in temperature, often manifests in behavioural and physiological responses like migration events, deposition of body fat and moulting^11,12^, hibernation^13,14^, torpor^15^ and more general alterations to activity patterns^16^. Mammals exhibit a variety of responses to a wide range of environmental conditions and thus provide an ideal model system on which to adopt macro-level approaches to the study of metabolic rate, both as an adaptation to different environments and life-histories.

Life histories frequently result in specific physiological adaptations and traits that are associated with a particular environment^17,18^. A number of factors have been found to influence BMR including diet, type of reproduction, and habitat, often interacting together in complex ways, leading to variation in physiological traits^19,20^. One other such example is living in confined spaces such as burrows and dens, where ambient oxygen levels are likely to be lower than outside^21^. This reduction in oxygen availability can present a challenge for species living in such environments. Despite the associated low oxygen levels, burrows, dens and crevices play an important role in the life history of many mammalian species^22^. Burrows can be used throughout the active period of the daily cycle, and/or as a safe retreat in which to sleep during the rest phase of the day. Moreover, they can be used seasonally to provide a place to safely raise young, or used more frequently as a place to live and possibly interact with conspecifics. Shelters create a locally contained environment that buffers the animal inside from external bioclimatic conditions, creating a microclimate around the individual or colony^21,23,24^.

Extreme adaptations in physiology, morphology and anatomy are evident in many species that exhibit a fossorial lifestyle. A lower BMR than similar sized closely related species has been observed in small mammalian species such as grey short-tailed opossums (*Monodelphis domestica*) and naked mole-rats (*Heterocephalus glaber*)^25,26^. These observations are suggested to be related to the respiratory stress hypothesis which proposes that low oxygen levels in burrows is thought to reduce gas exchange in oxygen-depleted environments and reduce heat storage. As a result, oxygen usage and metabolic rate are reduced^27–30^. This study will explore the potential impact on BMR of leading a fossorial lifestyle, and the effects on BMR of the degree of time spent underground. While the effects of hypoxia as a result of subterranean living have been studied in connection with energy expenditure and metabolism in individual species, particularly subterranean rodents, it has yet to be explored in a wider mammalian context. The aim of this macro-comparative phylogenetically informed study was to establish the effects of the use of burrows as a driver for low BMR in subterranean species in the broad context of the mammalian order. Due to the respiratory stress hypothesis, which suggests that hypoxic conditions found in burrows lead to poor gas exchange and thus lower metabolic rates^29,30^, we hypothesised that semi-fossorial and entirely fossorial species will exhibit lower BMRs than more surface-dwelling species, after accounting for body mass and phylogeny.

## Results

**Figure 1:**
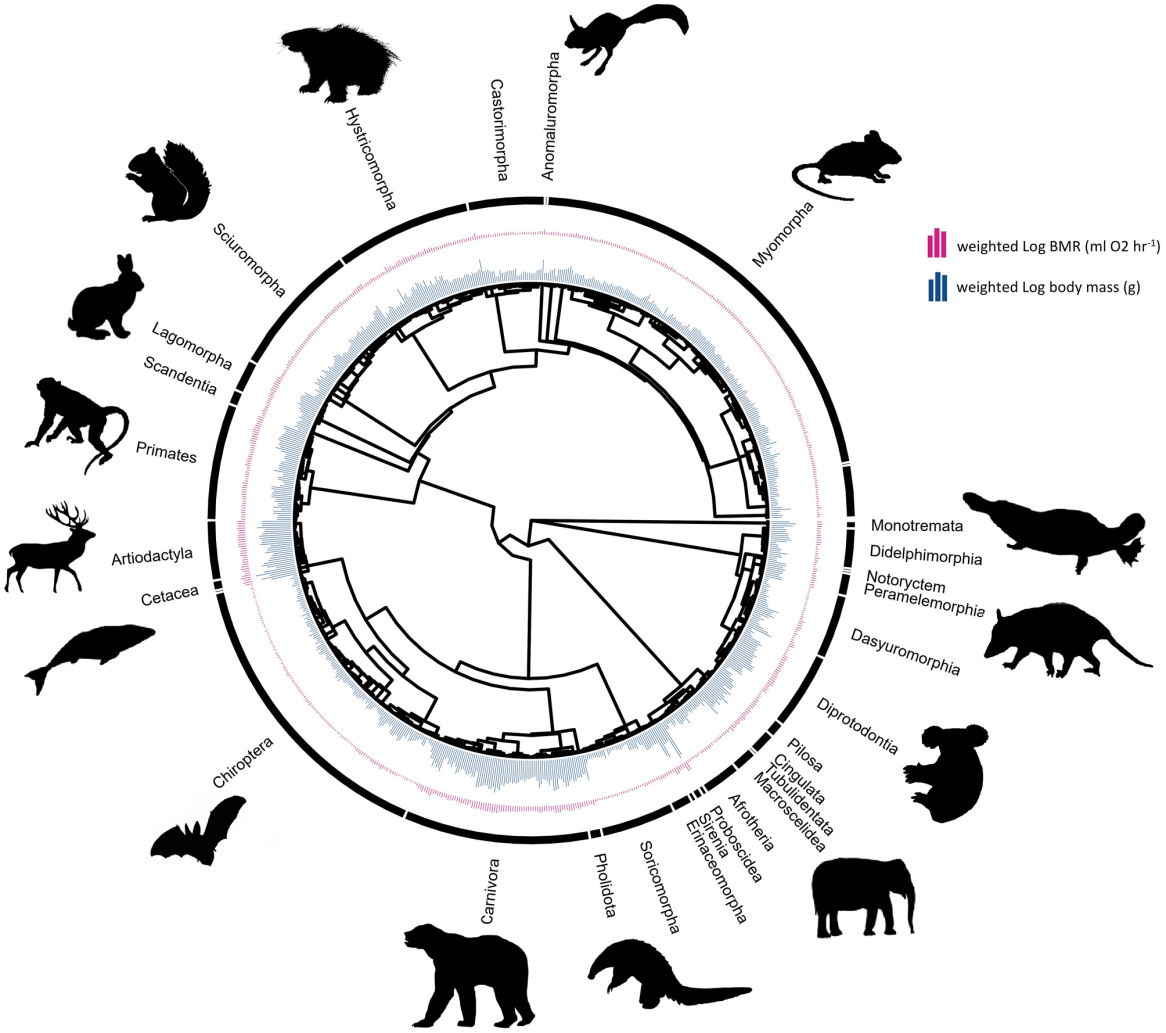
Phylogenetic tree showing all 801 mammalian species used in this study. The tree was obtained from the Open Tree of Life (OTL) and used in the PGLS and MCMCglmm. The inner bar plot represents weighted log body mass (g), and the outer bar plot represents weighted log BMR (ml O_2_ h^−1^). Mammalian order is shown in black around the phylogeny. BMR is strongly linked to phylogeny (Pagel’s λ; p < 0.001), and closely related species and clades will have similar BMRs compared to other individuals.

The phylogenetic least squares model (PGLS) was strongly influenced by phylogeny; the maximum likelihood estimate of Pagel’s Lambda (λ) was 0.95 for the top models. Pagel’s λ for the top models is significantly different from 0 (p < 0.001), which indicates that variation in BMR is associated more strongly with phylogeny rather than specific life history traits (Fig. 1). Thus, close relatives are likelier to have similar BMR than distant relatives.

**Figure 2:**
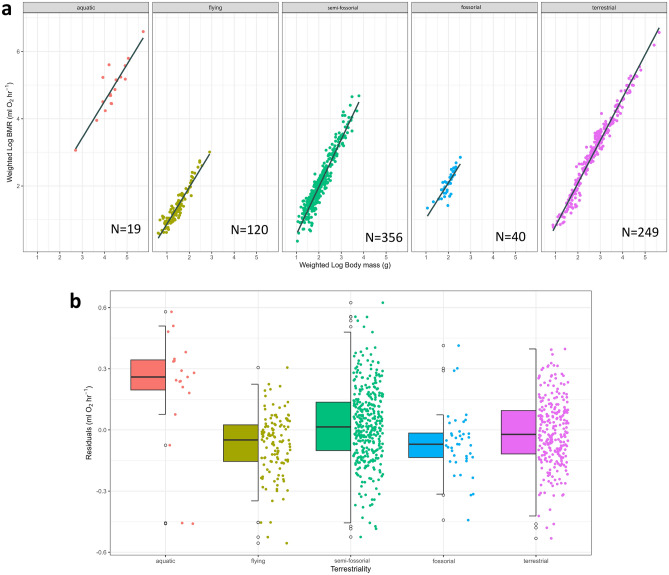
(**a**) Weighted log basal metabolic rate (BMR) (ml O_2_ h^−1^) against weighted log body mass (g) for each 801 mammal species in the study, across terrestrial categories. Regression lines are in black and confidence intervals are present in grey. Aquatic mammals demonstrated significantly higher BMRs for a given body mass than all other terrestrial groups, apart from ‘flying’ in the PGLS. No other groups were significantly different. (**b**) Box and jitter plot showing the residuals of weighted log basal metabolic rate (BMR) (ml O_2_ h^−1^) against weighted log body mass (g) across five terrestriality categories for all 801 mammal species used in this study.

The PGLS models established that the best-supported traits to explain variation in BMR are weighted log body mass (se = 0.01, *z* = 65.56, p < 0.05), annual temperature (se = 0.001, *z* = 2.71, p < 0.05), diurnal temperature range (se = 0.003, *z* = 3.45, p < 0.05), paternal care (se = 0.03, *z* = 3.05, p < 0.05) and isothermality (se = 0.0005, *z* = 2.46, p < 0.05) (Supplementary Table 5). BMR increased with body mass and decreased with increasing annual temperature, diurnal temperature range, isothermality and the presence of paternal care. Aquatic mammal BMR was found to be significantly higher than terrestrial (se = 0.07, *z* = 3.35, p < 0.05), semi-fossorial (se = 0.07, *z* = 3.26, p < 0.05) or fossorial (se = 0.08, *z* = 2.43, p < 0.05) categories (Fig. 2). All other traits were not retained in the final models (Supplementary Table 5).

**Figure 3:**
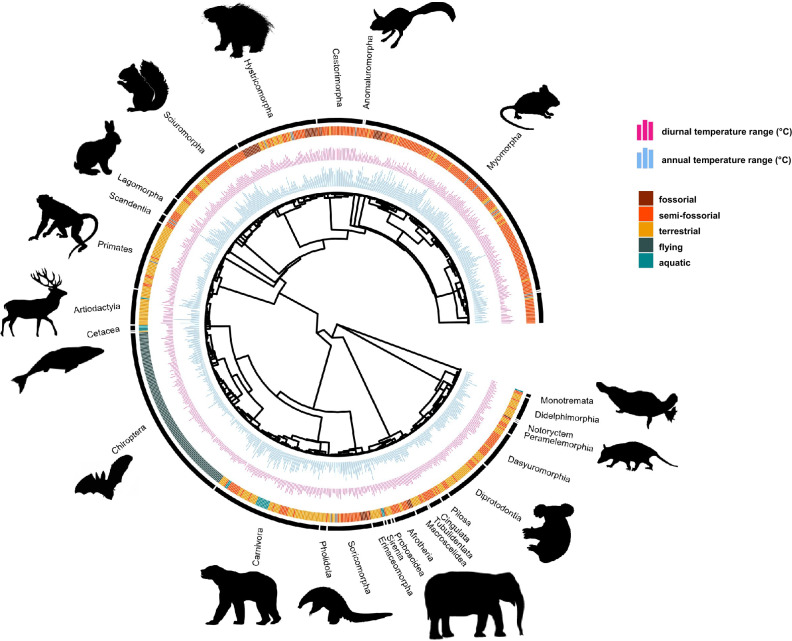
Phylogenetic tree showing all 801 mammalian species used in this study. The tree was obtained from OTL and used in the PGLS and MCMCglmm. The bar plots represent the significant predictors of BMR in the PGLS in conditionally averaged models for the PGLS and MCMCglmm. The inner bar plot represents annual temperature range (°C), and the outer bar plot represents diurnal temperature (°C). Terrestriality is colour coded and shown around the phylogeny, and mammalian order is shown in black.

The MCMCglmm established that weighted log body mass, annual temperature and diurnal temperature range were still significant even when taking into consideration intra-species variation (taking multiple studies per species into account) (Supplementary Table 6). Of the habitat categories, the aquatic category remained significantly different from the terrestrial, semi-fossorial and fossorial categories. Paternal care and isothermality were no longer retained as significant factors. Phylogenetic heritability (*H*^2^) was 0.95—close to 1—suggesting that BMR is strongly determined by phylogeny, and thus supporting the outcome of the PGLS (Fig. 3).

## Discussion

Overall, this study concludes that mammalian basal metabolic rate (BMR) was primarily driven by body mass and environmental temperature parameters; diurnal temperature and annual temperature range. Living underground was not found to influence BMR, and only aquatic mammals had a significantly increased BMR compared to terrestrial, fossorial and semi-fossorial species. A significantly lower BMR was found when paternal care was present, and as paternal care was shown to be a significant predictor trait in the PGLS but not the MCMCglmm, this suggests that elements of inter-specific variation, but not intra-specific variation, can be explained by paternal care of young.

Neither the semi-fossorial nor fossorial mammalian categories demonstrated a different BMR to terrestrial species and, therefore, there does not appear to be an associated BMR trace with living underground, either continuously or periodically. It is possible that species occupying a subterranean niche do not pay any baseline metabolic costs as a result of potential hypoxia and respiratory stress within burrows. This may facilitate certain species being able to occupy an underground shelter at night or during other periods of the day, whilst also spending significant amounts of time above ground. Moreover, it is possible that burrows may not be as physiologically challenging as previously suggested. Based on available data, burrow O_2_ and CO_2_ levels do not support the pronounced hypoxic conditions hypothesised in subterranean burrow systems^31^. Whilst further studies have demonstrated the pronounced hypoxia may not be as well established^31^, variation in soil type and structure, water content and animal activity may still influence gas concentrations in burrows at times. Furthermore, as the BMR measurements used in this analysis were likely carried out under normoxic conditions, and therefore will reflect BMR analysed under normothermic conditions, the values may not fully reflect the evolutionary adaptations of metabolic rate of an animal living in closed burrow systems with lower than ambient O_2_ levels.

Fossorial and semi-fossorial mammals, as well as aquatic mammals, experience periodic hypoxia that likely necessitates adaptations in basal physiology. Species that live for long periods in low hypoxic conditions may rely on phenotypic plasticity, which may be the case in both semi-fossorial and fossorial mammals. This means that to live in low O_2_ levels, BMR does not show a consistent directional adaptation, but rather there may be requirements for plasticity in metabolic rate, such as up-regulation or down-regulation of metabolism^16^. Furthermore, as per the respiratory stress hypothesis, individuals show morphological adaptations to enhance oxygen transfer from blood to metabolic active tissues, rather than physiological adaptations^29,30^. Hypoxia may determine maximal metabolism and this influence may not be detectable in measurements of BMR^32^. This contrasts with aquatic mammals that will experience conditions with limited oxygen for short bursts of time during dive events spanning 30 min to a few hours at a time^33^. These species will hold their breath in order to forage, socialise and move in water and are, therefore, experiencing periodic extremely low oxygen levels as pre-dive O_2_ stores are used during the dive, and CO_2_ accumulates^34^. It is possible that the difference between intensity and duration of exposure may result in a difference in metabolic traces. Species that are exposed regularly, but not continuously, to intense hypoxia likely have physiological mechanisms that they rely on to live in low oxygen conditions, such as increased oxygen affinity of haemoglobin molecules. Rapid short-term responses in metabolic rate have been observed in birds and mammals. Laughing doves (*Streptopelia senegalensis*) demonstrate short-term thermal acclimation capacities^35,36^, and when comparing two species of flycatchers, pied flycatchers (*Ficedula hypoleuca*) with a more variable metabolic rate demonstrated the ability to be more adaptive to climate change and spread their ranges further than collared flycatchers (*Ficedula albicollis*), which exhibited a more limited scope for metabolic change^37^. One species of subterranean rodent, *Spalacopus cyanus*, was exposed to low external temperatures in a laboratory experiment out of their normal range and, following repeated exposure, showed an increase in metabolic rate and thermogenic capacity in response to low temperatures, thus demonstrating acclimation^38^. Thus, it is possible that even in the case of species that are living entirely underground, physiology is still largely dictated by ambient temperatures above ground. Luna et al. demonstrated that ambient temperature plays a similar role in the evolution of residual BMR in terrestrial and subterranean rodents, suggesting that responses to modal ambient temperature are similar on a large scale, regardless of the thermal stability of the burrow systems^39^. The mechanistic process and how the temperature changes are sensed is still not very well understood. Short-term responses, instead of a permanent metabolic trace, may be important for both long-term and short-term energy management. There has been little to no research into what proportion of energy management is attributed to metabolic response to external and internal triggers, so it is unknown when and how much subterranean species may use this ‘activation to hypoxia’ response. As the hypothesis that hypoxic and hypercapnic conditions in burrow systems leads to respiratory stress^29^, the presence of a lower BMR even due to short-term exposure precludes hypoxia as an influencer of mammalian BMR. As such, thermal factors such as ambient surface and burrow temperatures appear to have a greater influence on BMR, and thermal physiological factors such as body temperature^39,40^. As climatic variables seem to be a greater predictor for metabolic rate, this shows that there may be an adaptive hierarchy, with the need to be adapted to the ambient temperature of their habitat being more important than the hypoxic conditions of their environment.

Climatic variables were shown to explain the observed differences between categories in BMR; annual temperature and diurnal temperature range were significant predictors retained in both the PGLS and the MCMCglmm models. Environmental temperature, therefore, seems to be a consistently significant predictor of variation in metabolic rate in this study. Climate may have an important influence on BMR due to most land-based mammals being comparatively more static in their movements across the land and in their home ranges^41^. This relative restriction in long-range movements compared to volant and aquatic mammals means that any changes in environmental temperature experienced will likely require physiological coping mechanisms due to the inability to evade unfavourable conditions. However, due to dominance of Chiropterans in the flying category it is not possible to separate whether the results of this group are influenced solely by phylogeny, and therefore no conclusions can be drawn from this. Mammals may utilise tactics such as hibernation and torpor. Species that do not employ these will likely need seasonal acclimations in BMR in response to these seasonal changes in the environment range^8–10^. Similarly, plasticity allows an organism to respond to changes in both daily and annual environmental conditions^16^. This, in turn, may also lead to changes in thermoneutral zones (TNZs), shifting to lower ranges in winter compared to summer, for example, reddish-grey mouse lemurs (*Microcebus griseorufus*) and striped hamsters (*Cricetulus barabensis*) have demonstrated adjustments to their TNZs in response to environmental variability in temperature, thus demonstrating that at large scales, ambient temperature can be associated with underground ambient temperature^41–44^.

Environmental temperature is an important determinant of BMR. Some mammalian species have very specific climatic adaptations and are, therefore, living in highly conserved temperature ranges such as Arctic foxes (*Vulpes lagopus*) and dromedary camels (*Camelus dromedarius*)^45,46^. Other mammals rely on very specific changes in temperatures and day length as triggers for hibernation, migration, and breeding^47–50^. Changes to the environmental temperatures that these species are experiencing are likely to have knock-on effects and alter population range changes, as well as the persistence of populations in certain areas. This highlights the importance of climate change and the large-scale effects that small changes in unseasonal environmental temperature may have on the diversity and distribution of mammalian species on the planet. It is important to take into consideration that sub-surface temperatures could not be obtained for the distribution ranges of the burrowing species used in this study. A future step would be to determine the sub-surface soil temperature at the correct burrowing depth of semi-fossorial and fossorial species to accurately quantify the temperatures experienced in the burrows and to further understand the impact of fossorial lifestyles on BMR.

Body mass was found to be a significant predictor of BMR. That adult body mass accounts for much of the variation in BMR has been well-established, where body mass increases so does BMR^51^. For the remaining, or residual variation, paternal care and isothermality were found to be significant predictors of mammalian BMR in the PGLS analyses but did not remain within the top MCMCglmms. Paternal care and isothermality may not influence BMR when taking into account variation between studies. The MCMCglmm may be used as a proxy for intra-species variation and could indicate that in this study, BMR varied enough within species to negate the effects on BMR.

This macro-comparative mammal study demonstrates that environmental temperature (annual temperature and diurnal temperature range) is an important determining factor when exploring differences in mammalian BMR and therefore strongly infers whether a mammal is physiologically suited to living in a particular place or habitat. There is no detectable trace in the BMR of mammalian species affiliated with living a subterranean lifestyle. This lack of a physiological trace suggests that hypoxia does not explain variation in BMR, but that inter-specific variation might play a role, and this variability may in part, explain why burrowing lifestyles evolve a number of times across multiple mammalian orders, all over the world.

## Methods

### Metabolic data

Data was collated from the literature, primarily using the dataset from Genoud et al.^52^. Genoud et al.^52^ created a highly quality controlled dataset to determine the value of BMR data and focussed on a methodological review of how basal metabolic rate (BMR; ml O_2_ h^−1^) is experimentally collected. In total, Genoud et al. collated the body mass and BMR measurements of 800 extant mammalian species across 30 taxonomic orders, including relevant information on the conditions under which the data were collected^52^. BMR has been debated with regard to its efficacy in drawing evolutionary conclusions, and a number of studies recommend the use of a number of physiological metrics in tandem^53,54^. However, BMR is still widely used, having been found to correlate with a range of ecological, physiological and life-history variables, as well as phylogeny^55–57^. Additional studies published since Genoud et al.^52^ were then added to the dataset for the present study. We searched for papers from 2015 to March 2021 using the search terms “mammal”, “BMR”, “RMR”, “basal metabolic rate” and “resting metabolic rate” on Google Scholar and Web of Science.

Essential inclusion criteria for our additional studies matched that of Genoud et al.^52^; fasted (post-absorptive), non-reproductive, adult, at thermoneutrality, during natural circadian resting phase, and at rest. Furthermore, we later limited inclusion to studies that had determined BMR using respirometry and additionally, only included BMR of non-domesticated species.

A total of 40 additional papers—published since 2018—were found to fit the criteria for inclusion in this study. If an aspect of the essential criteria was missing, contact was attempted via email to lead authors to obtain any missing information (N = 14). As with the Genoud et al.^52^ dataset, for the additional studies that did not fit the inclusion criteria, or where information regarding the criteria could not be obtained through email, these studies were not included. A total of nine authors replied with sufficient details so that the BMR values could then be included in the full data set and following this, 29 new BMR entries were added to the database. This consisted of 26 studies, assessing 28 species (across nine mammalian orders), nine species of which were novel in the database. In total, 801 mammal species across 1586 studies were used in the final dataset for this study.

### Life history trait data

Data on life history traits (LHTs) were selected based on known relevance and influence on BMR (Supplementary Tables 1, 2). The definitions for all life history traits collected were based on those outlined from the databases and books from which we obtained the relevant data (Supplementary Table 3)^58–66^. Life history traits included were: activity pattern, life span (months), brain mass (g), population density (/km^2^), annual number of litters, litter size, gestation (days), terrestriality, breeding seasonality, mating strategy, paternal care, upper elevation limit (m), diet and annual recruitment (/year). For the explanatory trait in the present study—terrestriality—the definitions and categories were outlined based on existing and accepted definitions, adjusting them where required to be more suitable in the context of mammals and the primary aim of the present study (Table 1). We included an intermediary group of species that are not obligately subterranean but do spend a significant amount of time in a burrow, den or crevice, which was termed semi-fossorial. This intermediate group provided the opportunity to detect if there was a potential gradient, and whether the species that fell into this group demonstrate a similar metabolic trace to the fossorial species. Definitions of terrestriality were initially tested through pilot data and allocation of definitions using 12 scientists at the start and end of the data collection to ensure that the definitions were robust, and to test for interobserver reliability in the use of these terms.

**Table 1:** List of definitions created for this study, to outline different terrestrial strategies: fossorial, semi-fossorial, terrestrial, flying and aquatic.

Terrestrial category	Definition
Fossorial—below ground only	Spends approx. whole life underground. Primarily digs/creates own closed burrow systems, which creates a controlled microclimate. Will show evidence of morphological and physiological adaptations to subterranean life, such as regression of eyesight or developed forelimbs adapted for digging
Semi-fossorial—below and on the ground	Spend significant time underground: using burrow at least once during a daily cycle (for sleeping, sheltering, eating, etc.). May create own burrow or may make use of existing burrows/crevices/cavities that are open or covered, but not completely closed. Some degree of a microclimate is created. May or may not hibernate
Terrestrial—on the ground only	Spends whole life above ground. Does not dig/create/make use of burrows or underground systems. Does not live in a controlled microclimate
Flying	Mammals capable of powered sustained flight. May or may not make use of cavities/crevices/caves but do not dig/create underground systems. Some degree of a microclimate may or may not be created
Aquatic	Mammals that are found predominantly in freshwater or marine environments. May or may not leave water for brief periods of time within a daily cycle. May or may not make use of cavities/crevices/caves but do not dig/create underground systems. Some degree of a microclimate may or may not be created

Explanations for any exceptions to the rule are also given. See Supplementary for Exceptions.

Numerous sources were accessed to obtain LHTs for all species for which we had BMR data. Where relevant—for continuous traits—a mean value was used as this was most readily available in databases and in books. In other cases, a maximum value was used as this again was most commonly found in databases. A detailed full list of sources can be found in the Supplementary Material (Supplementary Table 1). Any LHT with less than 60% of the data available was removed from the database and thus only nine of the initial 15 LHTs were retained (Supplementary Table 3).

Bioclimatic variables were obtained from the WorldClim—Global Climate Data website^67^ and were extracted using the ‘raster’ package in R version 4.1.1. The seven bioclimatic variables extracted were: annual precipitation (mm), precipitation seasonality (mm), annual temperature (°C), annual temperature range (°C), diurnal temperature range (°C), temperature seasonality (°C) and isothermality (°C) (Supplementary Table 3). This data was extracted at 2.5-min (of a longitude/latitude degree) spatial resolution (4.5 km at the equator)^67^. The median of each distinct geographical population for each species was extracted, and following this, the mean of the median was calculated for species that had more than one distinct geographic distribution.

### Statistical analysis

All calculations and statistical analyses were performed using the statistical software R version 4.1.1^68^.

### Obtaining BMR and body mass values

Log values for BMR (LogBMR) and body mass (LogBody mass) were used to account for the large range in the body mass of the species being used in this study (from 2.2 g (*Suncus etruscus*) to 4037.5 kg (*Orcinus orca*), and to reduce the skewness of the data^51^.

The database contained multiple studies per species in most cases, and therefore multiple associated BMR and body mass values at the species level. As multiple entries per species cannot be used in a phylogenetic least squares model (PGLS), a single mean value for both BMR and body mass was required. All 1586 studies in the final dataset were included, and for each of the 801 mammal species, where multiple studies per species were present a weighted average (for both BMR and body mass) was calculated. The larger the sample number used in a study, the more accurate the estimate^69^, so species mean values of BMR and body mass were weighted by the sample size for each study so that larger studies would contribute more to the overall mean value for a species. Thus, using LogBMR and LogBody mass, weighted basal metabolic rate (wLogBMR) and body mass (wLogBody mass) were obtained for inclusion in the PGLS.

### Co-linearity

A generalised variance inflation factor (GVIF) analysis, undertaken in a stepwise fashion, was used to determine multicollinearity between continuous and categorical LHTs. This identified three collinear traits (annual temperature range, temperature seasonality and annual recruitment), which were subsequently omitted from further analyses (Supplementary Table 4).

### PGLS

The database contained multiple studies per species; this duplication of species meant that a two-pronged approach was used. A PGLS was first used to determine the evolutionary association between BMR and the remaining LHTs within a phylogenetic context^70^. PGLS models were fitted using the phylolm function in the package ‘phylom’ and the ‘caper’ package^71,72^. All possible model combinations with up to five predictors were fitted using the pdredge function in package ‘MuMIn’^73^. AIC values were given, which measure the overall goodness of fit of each model, but not the significance of individual parameters^74^. All models with AIC values < 2 were retained and conditional model averaging then determined the importance of each trait present in these top ranking models^75^.

Pagel’s Lambda was estimated by maximum likelihood to evaluate the strength of the phylogenetic signal within the top models. This value ranges from 0 to 1 where 0 suggests no phylogenetic signal in the data and 1 indicates that phylogeny explains all the variation in mammalian BMR and is consistent with a Brownian motion model of trait evolution^76^.

### MCMCglmm

To confirm the results from the PGLS were robust, a generalised linear mixed-effects model using a Markov chain Monte Carlo approach under a Bayesian statistical framework (MCMCglmm) was applied in the ‘MCMCglmm’ package^77^. This approach was used to incorporate the multiple studies per species that were present in the database and thus account for intra-specific variation. A MCMCglmm was performed on the LogBMR for all 1586 studies in the database. This approach fits individual-level data whilst controlling for relationships in species traits due to common ancestry^78^. Phylogeny was included as a random effect within the mode based on mammalian order and species levels, to control for variation due to factors related to phylogeny. A single consensus tree was used, and 130,000 iterations were applied with 100 thinning intervals and 30,000 burn-in. In the main model, study-level BMR and body mass values were logged (LogBMR and Log body mass), thus producing one LogBMR and Log body mass value per study. Predicted BMR was calculated by regressing LogBMR against Log body mass. This was first run using the scaling coefficient for the whole dataset (0.74), and later a separate scaling coefficient for each terrestrial group was obtained to create unique predicted BMR values for each category (fossorial = 1.07, terrestrial = 1.28, semi-fossorial = 1.42, flying = 1.09 and aquatic = 1.11). The results from both did not differ significantly and the results presented are those obtained using the overall scaling coefficient.

LogBMR was set as a dependent variable, and independent variables were the significant predictor variable that were identified in the PGLS, namely Log body mass, annual temperature, diurnal temperature range, paternal care and terrestriality. Significance of the predictor variables carried forward from the PGLS were determined, and a heritability value (*H*^2^) were also obtained. The statistical significance of the genetic influence on BMR was assessed using 95% confidence intervals (CI) for the heritability estimates which is the transmission of the phenotypic variability within a population from generation to generation.

A mammalian phylogenetic tree was constructed in R using the package ‘rotl’. A taxonomic name resolution service (TNRS) was used to match taxon names in the database to the Open Tree Taxonomy identifiers. The Open Tree of Life (OTL) was used to create a final tree along with the backbone tree of the Open Tree Taxonomy (OTT). For replicability and standardisation reasons we have not altered the taxonomy of the tree. We carried out this analysis on 21/12/23 and the OTL and OTT reflect the mammalian phylogenetic tree as it was at this point in time.

### Ethics

All data was collected from pre-existing and published studies, therefore no permits or licenses were required for this study.

### Supplementary Information

Supplementary Information 1.

Supplementary Information 2.

Supplementary Information 3.

## Data Availability

All data are available as an Electronical Supplementary File.
