# Supplementary material for: No evidence for a signal in mammalian basal metabolic rate associated with a fossorial lifestyle
Source: Sci Rep. 2024 May 17;14:11297. doi: 10.1038/s41598-024-61595-1 (PMC11101413; doi:10.1038/s41598-024-61595-1)
Supplement: Supplementary file 1 — Supplementary Information 1. [file 41598_2024_61595_MOESM1_ESM.doc]

**SUPPLEMENTARY INFORMATION**

**Exceptions to Table 1**

Hibernating species and species that give birth and/or nurse young in burrows have been excluded from ‘semi-fossorial’ as it is defined by using a burrow at least once per daily cycle. The decision to exclude seasonal burrow users was made due to the likelihood of these species having seasonal physiological changes [1],[2],[3], potentially in order for them to occupy burrows, and it would not always be possible to ensure that studies obtained BMR measurements during this particular season or life-history stage. As such, the appropriateness of such BMR values could be questioned within the framework of the current study, and so were not included. Moreover, conditions for BMR are that individuals are not breeding, gestating or lactating, and thus species that nurse young in burrows do not fit our criteria, or that used for standard BMR measurements [4].

**Supplementary Table 1.** Table showing final life history traits, units and details of all sources used in this study.

| Life history and ecological traits | Unit of measure | Mean or max | Continuous or categorical | Source 1 | Source 2 | Source 3 | Source 4 |
| --- | --- | --- | --- | --- | --- | --- | --- |
| Activity pattern | N/A | N/A | categorical | [5] | [6] | [7] | [8] |
| Lifespan | months | max | continuous | [9] | [6] | [10] | [8] |
| Population density | /km2 | mean | continuous | [11] | [6] | N/A | [8] |
| Annual number of litters | N/A | mean | continuous | [11] | [6] | [12] | [8] |
| Litter size | N/A | mean | continuous | [11] | [6] | [12] | [8] |
| Gestation | days | mean | continuous | [6] | [11] | [13] | [8] |
| Terrestriality | N/A | N/A | categorical | [6] | [11] | [14],[15] | [8] |
| Breeding seasonality | N/A | N/A | categorical | [6] | [11] | [16],[17] | [8] |
| Paternal care | N/A | N/A | categorical | [18] | [6] | N/A | [8] |
| Upper elevational limit | m | max | continuous | [19] | [6] | [20] | [8] |
| Diet | N/A | N/A | categorical | [21] | [6] | [22],[23] | [8] |
| Annual temperature | °C | mean | continuous | [24] | N/A | N/A | N/A |
| Annual temperature range | °C | mean | continuous | [24] | N/A | N/A | N/A |
| Diurnal temperature range | °C | mean | continuous | [24] | N/A | N/A | N/A |
| Temperature seasonality | °C | mean | continuous | [24] | N/A | N/A | N/A |
| Isothermality | °C | mean | continuous | [24] | N/A | N/A | N/A |
| Annual precipitation | mm | mean | continuous | [24] | N/A | N/A | N/A |
| Precipitation seasonality | mm | mean | continuous | [24] | N/A | N/A | N/A |
| Annual recruitment | /yr | mean | continuous | = Annual number of litters x Litter size | N/A | N/A | N/A |
| Body mass | g | mean | continuous | Respective BMR studies | N/A | N/A | N/A |
| BMR | ml O2 hr-1 | mean | continuous | Respective BMR studies | N/A | N/A | N/A |

**Supplementary Table 2.** List of life history traits with units, rationale for inclusion and source of rationale.

| **Life history and ecological traits** | **Units** | **Relevance** | **Reference** |
| --- | --- | --- | --- |
| **Activity pattern** | N/A | Different activity levels require different energetic demands, and conditions differ between day, night and dusk, which may lead to differences in energetic requirements. | [25] |
| **Lifespan** | months | Longer lifespan is related to slower heart rate and therefore energetic demand. | [25] |
| **Brain mass** | g | Large brain mass means higher energetic demands for different brain sizes and potentially use more energy. | [26] |
| **Population density** | /km^2^ | The number of individuals within a given area relates to the quantity of resources required and provided, and thus the energetic demands individuals within the population. | [25] |
| **Annual number of litters** | N/A | Reproductive cycles place higher energetic demand on females. | [27] |
| **Litter size** | N/A | Large number of offspring, and associated provisioning, requires high energetic demand on females (and, more generally, parents) providing care for young. | [27] |
| **Gestation** | days | Longer gestation periods will require higher energetic demand for females. | [28] |
| **Terrestriality** | N/A | Living in a burrow mean accessibility to oxygen is reduced. Low oxygen environments require lower rate of metabolism so there is less oxygen required by the individual. | [29] |
| **Breeding seasonality** | N/A | Aseasonal breeding may result in high energetic demands to be reproductively active all year round as opposed to a set time of the year. | [30] |
| **Mating strategy** | N/A | Different mating strategies require different energetic demands for finding multiple mates, sperm competition, courtship and/or maintaining a single mate throughout a season or lifetime. | [31] |
| **Paternal care** | N/A | Care for young may mean a high energetic demand on parent(s). | [32] |
| **Upper elevational limit** | m | Higher elevation = lower oxygen levels in environment, may lower rate of metabolism. | [33] |
| **Diet** | N/A | Different diets have different foraging/hunting requirements and nutritional compositions, and therefore, different digestion processes which may vary in energetic demand. | [34] |
| **Annual precipitation** | mm | Precipitation related to aridity and could relate to differences in metabolic rate due to the differences in food and water requirements and heat production needed in different environments. | [35] |
| **Precipitation seasonality** | mm | Precipitation related to aridity and could relate to differences in metabolic rate due to the differences in food and water requirements and heat production needed in different environments. | [35] |
| **Annual temperature** | °C | Different temperatures may have different thermoregulatory demands to maintain a constant body temperature and therefore may affect metabolism. Annual temperatures consider the season changes in temperature which may have large variations, and differences in the amount of variation, depending on latitude. | [36] |
| **Annual temperature range** | °C | Different temperatures may have different thermoregulatory demands to maintain a constant body temperature and therefore may affect metabolism. Annual temperature range considers the extreme temperatures that species will experience and the range of temperatures they experience in a yearly cycle. | [36] |
| **Diurnal temperature range** | °C | Different temperatures may have different thermoregulatory demands to maintain a constant body temperature and therefore may affect metabolism. Temperatures change throughout the day being generally colder at night. | [36] |
| **Temperature seasonality** | °C | Different temperatures may have different thermoregulatory demands to maintain a constant body temperature and therefore may affect metabolism. Different seasons bring different climatic conditions which may affect vegetation growth and land use. | [36] |
| **Isothermality** | °C | Different temperatures may have different thermoregulatory demands to maintain a constant body temperature and therefore may affect metabolism. | [36] |
| **Annual recruitment** | /yr | Number of offspring/new recruits may have an effect on rate of metabolism due to demand of provisioning and overall increase in population resource demand. | [37] |

**Supplementary Table 3.** List of life history traits (LHTs) and their definitions, with associated references and sources. All source types are listed for each life history trait with final percentage species coverage of each trait. LHTs subsequently removed from study (see *Methods*) have their *‘% species coverage’* in bold. Two traits with <40% coverage were initially removed, then a further four traits with <60% species coverage were removed.

| **Life history traits** | **Definitions** | **Reference** | **Source description** | | | | **% species coverage** |
| --- | --- | --- | --- | --- | --- | --- | --- |
|  |  |  | **Source 1** | **Source 2** | **Source 3** | **Source 4** |  |
| **Activity pattern** | Regulation of activity over a 24-h sleep–wake cycle, concentrating peak periods of activity to coincide with the hours of daylight, darkness, or twilight, or using different periods of light and darkness in more complex ways. Nocturnal (active during night/darkness), diurnal (active during the day), crepuscular (active mostly during twilight), or cathemeral (active during hours of daylight and darkness). | [5] | Database from paper | Textbook | Primary literature | Textbook | 95 |
| **Brain mass** | The brain–body size allometry across all mammals is (Brain) = −1.26 (Body)^0.75^. | [38] | Database from paper | Textbook | Primary literature | Textbook | **32.9** |
| **Lifespan** | Duration of life from birth to death, in days. | [9] | Database from paper | Textbook | Primary literature | Textbook | **54.3** |
| **Population density** | Number of individuals in a population per square kilometre. | [11] | Website | Textbook | Primary literature | Textbook | **49.1** |
| **Annual number of litters** | Number of litters produced from the same mother, per year | [11] | Website | Textbook | Primary literature | Textbook | 67.6 |
| **Litter size** | Number of multiple offspring given birth to at one time, from the same mother. | [11] | Website | Textbook | Primary literature | Textbook | 91.2 |
| **Gestation** | Number of days between conception and birth during which an embryo develops. | [6] | Textbook | Website | Primary literature | Textbook | **40.7** |
| **Terrestriality** | See Table 1 | [6] | Textbook | Website | Primary literature | Textbook | 98 |
| **Breeding seasonality** | Breeding occurrence peaks throughout the year. Seasonal (occurring at a particular time of the year), aseasonal (generally 10ccurring at any time of the year). | [6] | Textbook | Website | Primary literature | Textbook | **46.7** |
| **Mating strategy** | Various techniques used by organisms to increase the likelihood that they produce offspring. | [6] | Textbook | Website | Primary literature | Textbook | **15.5** |
| **Paternal care** | A level of care to offspring provided by father. | [18] | Database from paper | Textbook | Primary literature | Textbook | 78.4 |
| **Upper elevational limit** | Highest elevation at which an individual of a species has been recorded. | [19] | Website | Textbook | Primary literature | Textbook | 99.5 |
| **Diet** | Resources that constitute the majority/entirety of a species food source. Herbivore (eats plant material only), carnivore (eats meat from animals/insects only), omnivore (eats both plant and animal material). | [21] | Database from paper | Textbook | Primary literature | Textbook | 65.1 |
| **Annual temperature** | Mean temperature in °C per year. | [24] | Website | NA | NA | NA | 99.5 |
| **Annual temperature range** | Range of lowest to highest temperatures per year. | [24] | Website | NA | NA | NA | 99.5 |
| **Diurnal temperature range** | Range of lowest to highest temperatures per day. | [24] | Website | NA | NA | NA | 99.5 |
| **Temperature seasonality** | Temperature change over the course of the year. | [24] | Website | NA | NA | NA | 99.5 |
| **Isothermality** | The day- to-night temperature oscillations relative to the summer- to-winter (annual) oscillations. | [24] | Website | NA | NA | NA | 99.5 |
| **Annual precipitation** | Mean precipitation in mm per year. | [24] | Website | NA | NA | NA | 99.5 |
| **Precipitation seasonality** | Precipitation change over the course of the year. | [24] | Website | NA | NA | NA | 99.5 |
| **Annual recruitment** | Annual number of litters x litter size. | NA | NA | NA | NA | NA | 66.8 |

**Supplementary Table 4.** Results of the stepwise VIF to remove continuous and categorical life history traits that are found to be colinear in this study. The trait with the highest GVIF is removed one by one until all traits GVIF are < 10, as in the final table. The trait being removed each time is in bold.

|  | **GVIF** | **Df** | **GVIF^(1/(2*Df))** |
| --- | --- | --- | --- |
| **Weighted log body mass** | 2.436014 | 1 | 1.560773 |
| **Activity pattern** | 1.894849 | 3 | 1.112404 |
| **Annual litters** | 5.729043 | 1 | 2.393542 |
| **Litter size** | 5.375436 | 1 | 2.318499 |
| **Paternal care** | 1.083809 | 1 | 1.041061 |
| **Diet** | 1.567089 | 2 | 1.118854 |
| **Annual recruitment** | 10.159856 | 1 | 3.187453 |
| **Annual temperature** | 9.255831 | 1 | 3.042340 |
| **Annual temperature range** | **233.284212** | **1** | **15.273644** |
| **Diurnal temperature range** | 27.300529 | 1 | 5.224991 |
| **Temperature seasonality** | 200.095133 | 1 | 14.145499 |
| **Isothermality** | 11.311646 | 1 | 3.363279 |
| **Annual precipitation** | 3.883379 | 1 | 1.970629 |
| **Precipitation seasonality** | 1.794499 | 1 | 1.339589 |
| **Terrestriality** | 3.945307 | 4 | 1.187162 |
| **Altitude** | 3.151923 | 1 | 1.775366 |

|  | **GVIF** | **Df** | **GVIF^(1/(2*Df))** |
| --- | --- | --- | --- |
| **Weighted log body mass** | 2.391337 | 1 | 1.546395 |
| **Activity pattern** | 1.875784 | 3 | 1.110530 |
| **Annual litters** | 5.728708 | 1 | 2.393472 |
| **Litter size** | 5.375125 | 1 | 2.318432 |
| **Paternal care** | 1.083530 | 1 | 1.040928 |
| **Diet** | 1.563792 | 2 | 1.118265 |
| **Annual recruitment** | 10.157318 | 1 | 3.187055 |
| **Annual temperature** | 9.240753 | 1 | 3.039861 |
| **Diurnal temperature range** | 4.859688 | 1 | 2.204470 |
| **Temperature seasonality** | **12.618597** | **1** | **3.552266** |
| **Isothermality** | 10.900904 | 1 | 3.301652 |
| **Annual precipitation** | 3.680505 | 1 | 1.918464 |
| **Precipitation seasonality** | 1.783173 | 1 | 1.335355 |
| **Terrestriality** | 3.870186 | 4 | 1.184313 |
| **Altitude** | 3.139577 | 1 | 1.771885 |

|  | **GVIF** | **Df** | **GVIF^(1/(2*Df))** |
| --- | --- | --- | --- |
| **Weighted log body mass** | 2.387867 | 1 | 1.545272 |
| **Activity pattern** | 1.860418 | 3 | 1.109009 |
| **Annual litters** | 5.680675 | 1 | 2.383417 |
| **Litter size** | 5.344148 | 1 | 2.311741 |
| **Paternal care** | 1.073040 | 1 | 1.035876 |
| **Diet** | 1.562357 | 2 | 1.118008 |
| **Annual recruitment** | **10.030817** | **1** | **3.167146** |
| **Annual temperature** | 6.482648 | 1 | 2.546104 |
| **Diurnal temperature range** | 3.396657 | 1 | 1.843002 |
| **Isothermality** | 5.757623 | 1 | 2.399505 |
| **Annual precipitation** | 3.516144 | 1 | 1.875138 |
| **Precipitation seasonality** | 1.758734 | 1 | 1.326173 |
| **Terrestriality** | 3.722680 | 4 | 1.178574 |
| **Altitude** | 2.750556 | 1 | 1.658480 |

|  | **GVIF** | **Df** | **GVIF^(1/(2*Df))** |
| --- | --- | --- | --- |
| **Weighted log body mass** | 2.339791 | 1 | 1.529637 |
| **Activity pattern** | 1.792638 | 3 | 1.102170 |
| **Annual litters** | 1.506310 | 1 | 1.227318 |
| **Litter size** | 1.745138 | 1 | 1.321037 |
| **Paternal care** | 1.068747 | 1 | 1.033802 |
| **Diet** | 1.518416 | 2 | 1.110063 |
| **Annual temperature** | 6.495359 | 1 | 2.548599 |
| **Diurnal temperature range** | 3.319045 | 1 | 1.821825 |
| **Isothermality** | 5.730607 | 1 | 2.393869 |
| **Annual precipitation** | 3.514720 | 1 | 1.874759 |
| **Precipitation seasonality** | 1.726898 | 1 | 1.314115 |
| **Terrestriality** | 3.577995 | 4 | 1.172749 |
| **Altitude** | 2.750325 | 1 | 1.658411 |

**Supplementary Table 5.** Phylogenetic least squares model (PGLS) of basal metabolic rate (ml O_2_ hr^-1^) on 801 mammal species including a number of life history traits (LHTs) that may influence BMR. LHTs with a p<0.05 are marked with an asterisk and best supported the observed variation in log BMR.

|  | **Estimate** | **Std Error** | ***Z* value** | **Pr(>|z|)** |
| --- | --- | --- | --- | --- |
| **(Intercept)** | 0.87 | 0.23 | 3.8 | 0.00015 |
| **Weighted log body mass** | 0.75 | 0.01 | 65.56 | <0.01* |
| **Annual temperature** | -0.003 | 0.001 | 2.71 | 0.007* |
| **Diurnal temperature range** | -0.01 | 0.003 | 3.45 | <0.01* |
| **Paternal care: no~yes** | -0.1 | 0.03 | 3.05 | <0.01* |
| **Isothermality** | -0.001 | 0.0005 | 2.46 | 0.01* |
| **Annual litters** | 0.01 | 0.01 | 0.91 | 0.36 |
| **Annual precipitation** | <0.01 | <0.01 | 0.81 | 0.42 |
| **Annual recruitment** | <0.01 | <0.01 | 0.38 | 0.70 |
| **Aquatic~flying** | -0.32 | 0.2 | 1.65 | 0.10 |
| **Aquatic~semi-fossorial** | -0.22 | 0.07 | 3.26 | <0.01* |
| **Aquatic~fossorial** | -0.2 | 0.08 | 2.43 | 0.02* |
| **Aquatic~terrestrial** | -0.22 | 0.07 | 3.35 | <0.01* |
| **Flying~semi-fossorial** | 0.12 | 0.18 | 0.66 | 0.51 |
| **Flying~fossorial** | 0.13 | 0.18 | 0.69 | 0.49 |
| **Flying~terrestrial** | 0.12 | 0.18 | 0.66 | 0.51 |
| **Terrestrial~semi-fossorial** | <0.01 | 0.02 | 0.01 | 0.99 |
| **Terrestrial~fossorial** | 0.01 | 0.05 | 0.20 | 0.84 |
| **Semi-fossorial~semi-fossorial** | -0.01 | 0.05 | 0.21 | 0.84 |
| **Crepuscular~cathemeral** | 0.03 | 0.05 | 0.57 | 0.57 |
| **Crepuscular~diurnal** | 0.02 | 0.03 | 0.55 | 0.58 |
| **Crepuscular~nocturnal** | 0.01 | 0.03 | 0.36 | 0.72 |
| **Cathemeral~diurnal** | -0.01 | 0.03 | 0.39 | 0.70 |
| **Cathemeral~nocturnal** | -0.05 | 0.03 | 1.90 | 0.06 |
| **Diurnal~nocturnal** | -0.04 | 0.02 | 1.73 | 0.09 |

**Supplementary Table 6.** MCMCglmm for top 4 models from PGLS for all 1,586 studies across 801 species. The life history traits that were significant in the PGLS were carried forward and significance was tested when taking into account multiple studies per species and therefore intra-species variation.

|  | **post.mean** | **l-95% CI** | **u-95% CI** | **eff.samp** | **pMCMC** |
| --- | --- | --- | --- | --- | --- |
| **(Intercept)** | 0.77 | 0.29 | 1.22 | 756.8 | 0.004 |
| **Log body mass** | 0.73 | 0.71 | 0.74 | 764.6 | <0.001 |
| **Annual temperature** | -0.002 | -0.003 | -0.0008 | 1198.1 | <0.001 |
| **Diurnal temperature range** | -0.008 | -0.012 | -0.004 | 1122.1 | <0.001 |
| **Paternal care: no~yes** | -0.035 | -0.07 | 0.006 | 1000.0 | 0.11 |
| **Aquatic~flying** | -0.38 | -0.87 | 0.03 | 852.3 | 0.08 |
| **Aquatic~semi-fossorial** | -0.22 | -0.30 | -0.14 | 1000.0 | <0.001 |
| **Aquatic~fossorial** | -0.22 | -0.33 | -0.11 | 895.9 | <0.001 |
| **Aquatic~terrestrial** | -0.23 | -0.3 | -0.15 | 1000 | <0.001 |
| **Flying~semi-fossorial** | 0.17 | -0.26 | 0.58 | 1000 | 0.43 |
| **Flying~fossorial** | 0.17 | -0.26 | 0.58 | 1000 | 0.44 |
| **Flying~terrestrial** | 0.16 | -0.24 | 0.59 | 1000 | 0.43 |
| **Terrestrial~semi-fossorial** | 0.004 | -0.02 | 0.03 | 1126.8 | 0.79 |
| **Terrestrial~fossorial** | -0.0005 | -0.09 | 0.08 | 1000 | 0.99 |
| **Semi-fossorial~fossorial** | 0.0006 | -0.09 | 0.07 | 1000 | 0.97 |


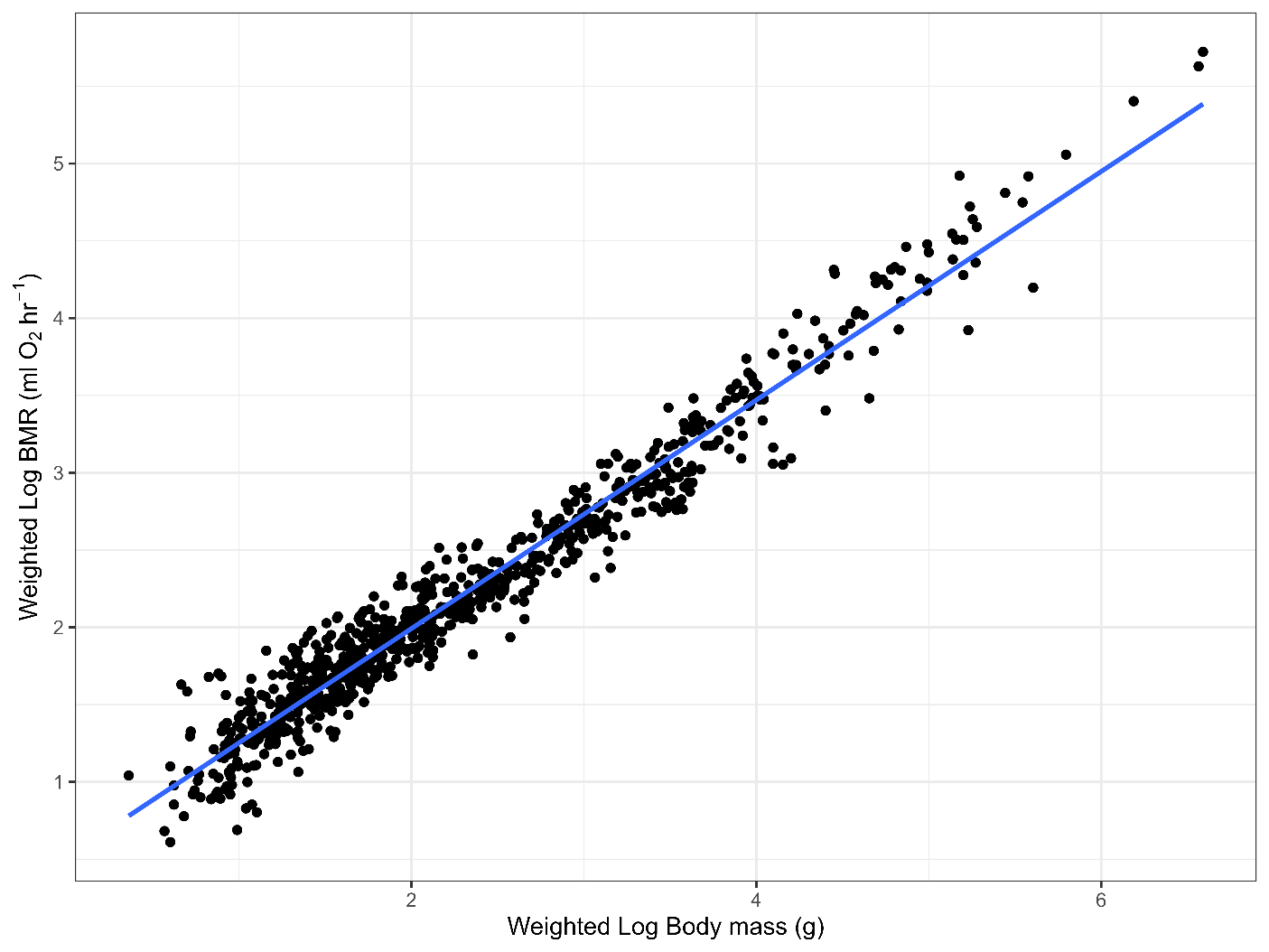


**Supplementary Figure 1.** Weighted log basal metabolic rate (BMR) (ml O_2_ hr^-1^) against weighted log body mass (g) for 801 mammal species. Regression line is shown in blue. Confidence intervals are present in grey.


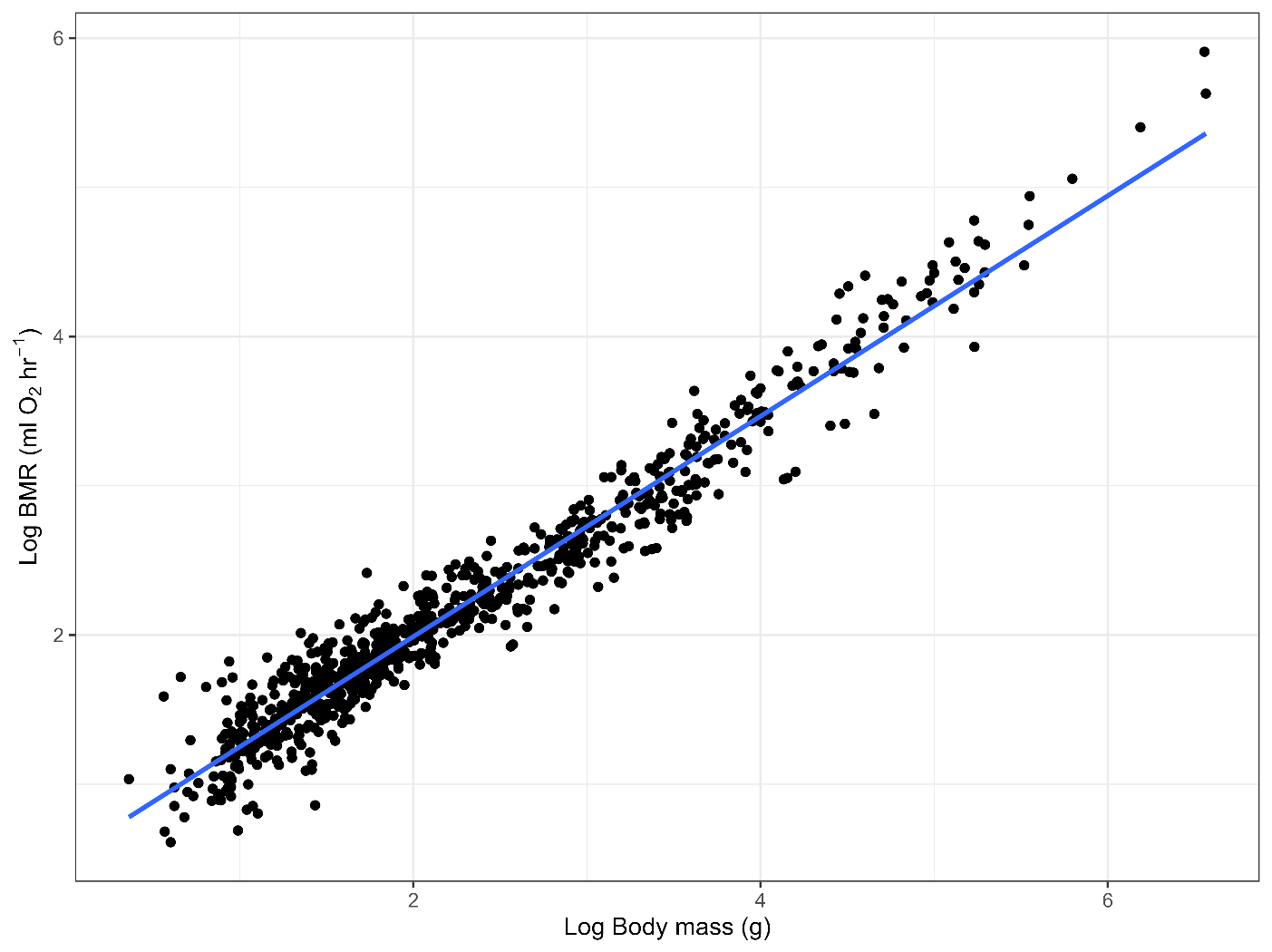


**Supplementary Figure 2.** Log Basal metabolic rate (BMR) (ml O_2_ hr^-1^) against log Body mass (g) for all 1586 studies (N = 801 mammal species) used in this study.

**REFERENCES**

1. Frenche, A.R. (1985). Allometries of the durations of torpid and euthermic intervals during mammalian hibernation: a test of the theory of metabolic control of the timing of changes in body temperature. Journal of Comparative Physiology B, 156(1), pp.13-19.
2. Carey, H.V., Walters, W.A. and Knight, R. (2013). Seasonal restructuring of the ground squirrel gut microbiota over the annual hibernation cycle. American Journal of Physiology-Regulatory, Integrative and Comparative Physiology, 304(1), pp.33-42.
3. Morrow, G.E. and Nicol, S.C. (2013). Maternal care in the Tasmanian echidna (Tachyglossus aculeatus setosus). Australian Journal of Zoology, 60(5), pp.289-298.
4. Halsey, L.G. (2011). The challenge of measuring energy expenditure: current field and laboratory methods. Comparative Biochemistry and Physiology. Part A, Molecular & Integrative Physiology, 158(3), pp.247–251.
5. Bennie, J.J., Duffy, J.P., Inger, R. and Gaston, K.J. (2014). Biogeography of time partitioning in mammals. Proceedings of the National Academy of Sciences, 111(38), pp.13727-13732.
6. Kleiman, D.G., Geist, V. and McDade, M.C. (2003). Grzimek’s animal life encyclopedia. Mammals I-IV, Gale, Detroit, MI.
7. Pietersen, D.W., Jansen, R., Swart, J., Panaino, W., Kotze, A., Rankin, P. and Nebe, B., 2020. Temminck’s pangolin Smutsia temminckii (Smuts, 1832). Pangolins: Science, Society and Conservation. Elsevier, Amsterdam, pp.175-193.
8. Wilson, D.E., and Mittermeier, R.A. (2011). Handbook of the mammals of the world. Lynx Ediciones, Barcelona, Spain.
9. DeCasien, A.R. Thompson, N.A., Williams, S.A. and Shattuck, M.R., (2018). Encephalization and longevity evolved in a correlated fashion in Euarchontoglires but not in other mammals. Evolution, 72(12), pp.2617-2631.
10. Dammann, P., Šumbera, R., Maßmann, C., Scherag, A. and Burda, H. (2011). Extended longevity of reproductives appears to be common in Fukomys mole-rats (Rodentia, Bathyergidae). PLoS One, 6(4), p.e18757.
11. Parr, C.S., Wilson, M.N., Leary, M.P., Schulz, K.S., Lans, M.K., Walley, M.L., Hammock, J.A., Goddard, M.A., Rice, M.J., Studer, M.M. and Holmes, J.T. (2014). The encyclopedia of life v2: providing global access to knowledge about life on earth. Biodiversity Data Journal, (2), pp.10-79.
12. Frynta, D., Fraňková, M., Čížková, B., Skarlandtová, H., Galeštoková, K., Průšová, K., Šmilauer, P. and Šumbera, R. (2011). Social and life history correlates of litter size in captive colonies of precocial spiny mice (Acomys). Acta Theriologica, 56, pp.289-295.
13. Atramentowicz, M. (1995). Growth of pouch young in the bare-tailed woolly opossum, Caluromys philander. Journal of mammalogy, 76(4), pp.1213-1219.
14. Deacon, R.M.J. (2009). Burrowing: a sensitive behavioural assay, tested in five species of laboratory rodents. Behavioural Brain Research, 200(1), pp.128-133.
15. Karasov, W.H. (1992). Daily energy expenditure and the cost of activity in mammals. American Zoologist, 32(2), pp.238-248.
16. Herbst, M., Jarvis, J.U.M. and Bennett, N.C. (2004). A field assessment of reproductive seasonality in the threatened wild Namaqua dune mole-rat (Bathyergus janetta). Journal of Zoology, 263(3), pp.259-268.
17. El Bizri, H.R., Fa, J.E., Bowler, M., Valsecchi, J., Bodmer, R. and Mayor, P. (2018). Breeding seasonality in the lowland paca (Cuniculus paca) in Amazonia: interactions with rainfall, fruiting, and sustainable hunting. Journal of Mammalogy, 99(5), pp.1101-1111.
18. Stockley, P. and Hobson, L. (2016). Paternal care and litter size coevolution in mammals. Proceedings of the Royal Society B: Biological Sciences, 283(1829), pp.1471-2954.
19. IUCN 2020. The IUCN Red List of Threatened Species. Version (2020-3). https://www.iucnredlist.org. Downloaded on [01/05/20].
20. Price, M.V. (1978). The role of microhabitat in structuring desert rodent communities. Ecology, 59(5), pp.910-921.
21. Tucker, M.A., Ord, T.J. and Rogers, T.L. (2014). Evolutionary predictors of mammalian home range size: body mass, diet and the environment. Global Ecology and Biogeography, 23(10), pp.1105-1114.
22. Kuyper, M.A. (1985). The ecology of the golden mole Amblysomus hottentotus. Mammal Review, 15(1), pp.3-11.
23. Bilenca, D.N. and Kravetz, F.O. (1998). Seasonal variations in microhabitat use and feeding habits of the pampas mouse Akodon azarae in agroecosystems of central Argentina. Acta Theriologica, 43, pp.195-204.
24. Fick, S.E. and Hijmans, R.J. (2017). WorldClim 2: new 1‐km spatial resolution climate surfaces for global land areas. International Journal of Climatology, 37(12), pp.4302-4315
25. White, C.R. and Seymour, R.S. (2004). Does basal metabolic rate contain a useful signal? Mammalian BMR allometry and correlations with a selection of physiological, ecological, and life-history variables. Physiological and Biochemical Zoology, 77(6), pp.929-941.
26. Isler, K. and Van Schaik, C.P. (2006). Metabolic costs of brain size evolution. Biology Letters, 2(4), pp.557-560.
27. Kenagy, G.J., Masman, D., Sharbaugh, S.M. and Nagy, K.A. (1990). Energy expenditure during lactation in relation to litter size in free-living golden-mantled ground squirrels. The Journal of Animal Ecology, pp.73-88.
28. Thompson, S.D. and Nicoll, M.E. (1986). Basal metabolic rate and energetics of reproduction in therian mammals. Nature, 321(6071), pp.690-693.
29. McNab, B.K. (1966). The metabolism of fossorial rodents: a study of convergence. Ecology, 47(5), pp.712-733.
30. Piersma, T., Cadée, N. and Daan, S. (1995). Seasonality in basal metabolic rate and thermal conductance in a long-distance migrant shorebird, the knot (Calidris canutus). Journal of Comparative Physiology B, 165(1), pp.37-45.
31. Tourmente, M., Gomendio, M. and Roldan, E.R. (2011). Mass-specific metabolic rate and sperm competition determine sperm size in marsupial mammals. PLoS One, 6(6), pp.44.
32. Farmer, C.G. (2000). Parental care: the key to understanding endothermy and other convergent features in birds and mammals. The American Naturalist, 155(3), pp.326-334.
33. Hayes, J.P. (1989). Altitudinal and seasonal effects on aerobic metabolism of deer mice. Journal of Comparative Physiology B, 159(4), pp.453-459.
34. Ross, C. (1992). Basal metabolic rate, body weight and diet in primates: an evaluation of the evidence. Folia Primatologica, 58(1), pp.7-23
35. Tieleman, B.I., Williams, J.B. and Bloomer, P. (2003). Adaptation of metabolism and evaporative water loss along an aridity gradient. Proceedings of the Royal Society of London. Series B: Biological Sciences, 270(1511), pp.207-214.
36. White, C.R., Blackburn, T.M., Martin, G.R. and Butler, P.J. (2007). Basal metabolic rate of birds is associated with habitat temperature and precipitation, not primary productivity. Proceedings of the Royal Society B: Biological Sciences, 274(1607), pp.287-293.
37. Rønning, B., Broggi, J., Bech, C., Moe, B., Ringsby, T.H., Pärn, H., Hagen, I.J., Sæther, B.E. and Jensen, H. (2016). Is basal metabolic rate associated with recruit production and survival in free‐living house sparrows?. Functional Ecology, 30(7), pp.1140-1148.
38. Burger, J.R., George Jr, M.A., Leadbetter, C. and Shaikh, F. (2019). The allometry of brain size in mammals. Journal of Mammalogy, 100(2), pp.276-283.
